# Families as catalysts for peer adherence support in enhancing hope for people living with HIV/AIDS in South Africa

**DOI:** 10.7448/IAS.17.1.18802

**Published:** 2014-04-03

**Authors:** Caroline Masquillier, Edwin Wouters, Dimitri Mortelmans, Frederik le Roux Booysen

**Affiliations:** 1 Department of Sociology, Research Centre for Longitudinal and Life Course Studies (CELLO), University of Antwerp, Antwerp, Belgium; 2 Centre for Health Systems Research and Development, University of the Free State, Bloemfontein, South Africa; 3 Department of Economics, University of the Free State, Bloemfontein, South Africa

**Keywords:** peer adherence support, hope, family functioning, latent cross-lagged modelling, randomized controlled trial, South Africa

## Abstract

**Introduction:**

Hope is an essential dimension of successful coping in the context of illnesses such as HIV/AIDS, because positive expectations for the future alleviate emotional distress, enhance quality of life and have been linked to the capacity for behavioural change. The social environment (e.g. family, peers) is a regulator of hope for people living with HIV/AIDS (PLWHA). In this regard, the dual aim of this article is (1) to analyze the influence of a peer adherence support (PAS) intervention and the family environment on the state of hope in PLWHA and (2) to investigate the interrelationship between the two determinants.

**Methods:**

The *Effective AIDS Treatment and Support in the Free State* study is a prospective randomized controlled trial. Participants were recruited from 12 public antiretroviral treatment (ART) clinics across five districts in the Free State Province of South Africa. Each of these patients was assigned to one of the following groups: a control group receiving standard care, a group receiving additional biweekly PAS or a group receiving PAS and nutritional support. Latent cross-lagged modelling (Mplus) was used to analyse the impact of PAS and the family environment on the level of hope in PLWHA.

**Results:**

The results of the study indicate that neither PAS nor the family environment has a direct effect on the level of hope in PLWHA. Subsequent analysis reveals a positive significant interaction between family functioning and PAS at the second follow-up, indicating that better family functioning increases the positive effect of PAS on the state of hope in PLWHA.

**Conclusions:**

The interplay between well-functioning families and external PAS generates higher levels of hope, which is an essential dimension in the success of lifelong treatment. This study provides additional insight into the important role played by family dynamics in HIV/AIDS care, and it underscores the need for PAS interventions that are sensitive to the contexts in which they are implemented.

## Introduction

After many years of disappointment, the international community was filled with hope in 1996, when scientists were able to report a significant treatment breakthrough: highly active antiretroviral therapy (HAART) [1,2]. This spirit was reflected in the theme of the 11th World AIDS Conference: “One World, One Hope” [1,3]. At the international level, access to treatment has generated overarching narratives of hope and promise regarding the possibility of mitigating the effects of the HIV epidemic [3–5]. A major effort to scale-up antiretroviral therapy (ART) was introduced in 2003 [6]. In South Africa, the *Operational Plan for Comprehensive HIV and AIDS Care, Management and Treatment for South Africa* was launched in November 2003, with the goal of initiating ART nationally [7,8]. In addition to saving lives, increasing adult life expectancy [9] and having a population-level preventive effect [10], the ART scale-up greatly enhanced staff morale [4,11]. As articulated by Kazatchinke (2008), “hope for millions of people in the developing world affected by HIV is no longer utopia but evidence based” [Kazatchinke in 3]. Hope is an essential dimension of successful coping in the context of HIV/AIDS [12–15], because positive expectations for the future alleviate emotional distress [16], enhance quality of life [17] and have been linked to the capacity for behavioural change [14,18,19].

**Figure 1:**
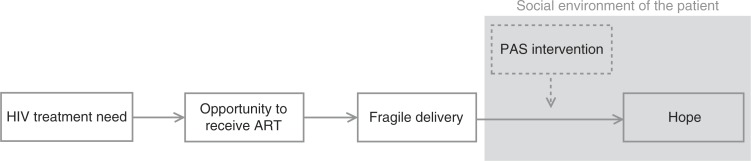
The HIV/AIDS epidemic: ART and hope. Source: Adaptation based on [18].

Bernays, Rhodes and Barnett (2007) provide a conceptual framework for understanding hope in relation to HIV treatment, a portion of which is presented in Figure 1. The model draws attention to the uneasy balance between access expectations and the lived experiences of treatment delivery within fragile health systems. These authors warn that such environmental conditions “may puncture or limit hope, leading to a lack of investment in the future as realized through engagement in HIV prevention and HIV treatment” [18]. Despite the major scale-up of ART, challenges remain with regard to ART in high-burden countries, due to the weakness of their health systems and absorptive capacity [20,21]. For example, the Free State province is characterized by a shortage of health care workers, particularly doctors. The ART programme in this province is, therefore, driven primarily by nurses [4,22]. Additional human resources are required in order to develop a sustainable treatment strategy [20]. As estimated by Hontelez and colleagues [23], an additional 2200 nurses, 3800 counsellors and 300 doctors would be required in order to achieve universal access to HIV treatment for all patients with a CD4 cell count of ≤350 cells/µL in South Africa [23]. In addition to this structural shortcoming in the health care system, the Free State province has also encountered temporary challenges. From November 2008 until February 2009 – the period during which this survey was conducted – the Department of Health in the Free State province stopped initiating ART for new patients because of out-of-stock drugs and a lack of funds [24]. A four-month provincial moratorium barred new patients from receiving ART, resulting in a waiting list of over 15,000 people [25]. In addition to increased morbidity and mortality during the ART stock-out, researchers reported a loss of trust in the health care system and anxiety about this system's ability to guarantee lifelong access to ART [24].

Long-term success in the prevention and treatment of HIV requires an environment that protects hope. Without creating an illusion of hope through global promises that cannot be met locally, it is important to invest in social and structural interventions to create such an environment [18]. As suggested by Bernays, Rhodes and Barnett [3,18], psychosocial support for those delivering, receiving and waiting for treatment could be an appropriate intervention within local settings. In the context of human-resource shortages, studies are increasingly exploring the potential benefits of peer supporters in providing psychosocial care [26–28]. Being around and receiving support from others who are experiencing similar circumstances can help people living with HIV/AIDS (PLWHA) to experience hope [13,14]. In addition to peer supporters, the family has been identified as an important source of hope for those learning about their HIV-positive status [14,29], as well as for sustaining this hope when living with HIV/AIDS for longer periods [29,30]. The social environment in which PLWHA live is thus a regulator of hope [18,31]. The socio-ecological perspective emphasizes the interrelatedness and interdependency of individuals and their environments. In this regard, PLWHA are in continuous interaction with their social environments, which influences their level of hope [32–35].

As suggested by the conceptual framework developed by Bernays, Rhodes and Barnett [18], there is a need for knowledge regarding how to enhance hope within the context of fragile delivery, as shown in Figure 1. In this regard, the current study has two related objectives: (1) to analyse the impact of peer adherence support (PAS) intervention and the family environment on the state of hope in PLWHA in the Free State and (2) to investigate the interrelationship between this type of intervention and the immediate social context in which a patient lives.

## Methods

### Setting

This study is part of a prospective cohort study entitled *Effective AIDS Treatment and Support in the Free State* (FEATS), which was conducted by the Centre for Health Systems Research and Development at the University of the Free State (UFS). The study was approved by the Ethics Committee of the UFS Faculty of Health Sciences [ETOVS 145/07], and it is registered in South Africa National trial register [DOH-27-0907-2025], as well as with the National Institutes of Health in the United States [NCT00821366]. Written informed consent was obtained from study participants using a standardized questionnaire process before the interview. Participants consented to participate in the randomized controlled trial, in addition to allowing researchers to access their patient files during the collection of clinical data.

### Sample

Participants in the study were recruited from 12 public ART clinics across five districts in the Free State Province of South Africa (i.e. Lejweleputswa; Motheo; Thabo Mofutsanyana; Fezile Dabi; Xhariep), based on the following inclusion criteria: 18 years of age or older, residing in the town or village in which the particular health care facility is located, and having initiated ART in the past month given that the primary research aim was to observe patients early in their treatment careers and prior to achieving complete clinical response to ARV treatment [36]. Clinical data were obtained by accessing patient files and electronic data from the National Health Laboratory Services Data. The median time between two consecutive interviews was 11.7 months. The baseline data collection (approximately one month after initiating ART) for this longitudinal survey was conducted from October 2007 until October 2008 amongst 653 PLWHA. The second wave of data collection took place from April until October 2009, resulting in 498 completed interviews. The third and final round of interviews was held from March until June 2010, with 422 participants. Attrition was primarily due to mortality among study participants (42.4%) and unknown whereabouts (34.1%) [37], with no statistically differential attrition occurring as a function of the study condition.

### PAS intervention in the Free State province

The primary research aim of this randomized controlled trial is to investigate the effectiveness of AIDS treatment and support in settings where free ART has already been introduced. In this regard, all patients received ART and the associated support provided in the public sector ART programme, as published in the National Treatment Guidelines for ART of Adults [38]. To qualify for the initiation of ART, patients were required to meet the following criteria: (1) having a CD4 cell count lower than 200 cells/m^3^ regardless of stage; or (2) WHO Stage IV AIDS-defining illness irrespective of CD4 count; and (3) expressing willingness and readiness to adhere to ART. The following treatment regimens were used in the public service: regimens 1a (d4T/3TC/efavirenz), 1b (d4T/3TC/NVP), and 2 (AZT/ddI/lopinavir/ritonavir). Following the baseline survey, patients recruited into the study were randomly assigned either to a control group or to a group receiving additional bi-weekly PAS for a period of 18 months [39]. Assignment was performed according to a Zelen-type double-randomized consent design. A subset of the patients receiving PAS also received nutritional support (i.e. two 400 g cans of spaghetti and meatballs in tomato sauce). The peer adherence supporters were PLWHA who had been on ART for at least 12 months and who had received a theoretical and practical training on HIV/AIDS, ART and adherence, nutrition and infection control in the home, based on material developed by the UFS's School of Nursing. When visiting patients, peer adherence supporters provided support with adherence and discussed matters that can make adherence more difficult (e.g. stigma). They identified possible side effects of ART and acted appropriately. When necessary, they referred patients to a clinic. Other topics (e.g. unemployment or pension grants) were discussed as well. The meetings took place at a time and place specified by the PLWHA, whether in the home, at work or elsewhere [36].

With regard to the primary research aim, intent-to-treat analysis of the FEATS data has shown that PAS has a significant impact on CD4 counts. The CD4 counts of patients assigned to the PAS arm of the experiment (regardless of whether they received additional nutritional support) were, on average, 32.5/µL higher than those of patients in the control group [39]. Although no specific intervention content targeted hope, we expect it to have a spillover effect on the level of hope in addition to the effect of the PAS intervention on this primary research outcome. This expectation is based on the aforementioned theoretical foundation developed by Bernays, Rhodes and Barnett [18].

### Measures

Confirmatory factor analysis was used to examine the latent structure of hope and family functioning. In order to evaluate whether the resulting parameter estimates were good measures of their latent constructs, we included items with factor loadings above the 0.40 threshold [40]. Following the method described by Hatcher (1994), the composite reliability of the scales was calculated. A score above 0.70 indicates a reliable scale [41].

#### Hope

The Adult State of Hope Scale developed by Snyder and colleagues [42,43] was used, based on Snyder's definition of hope as “a positive motivational state that is based on an interactively derived sense of successful (a) agency (goal-directed energy) and (b) pathways (planning to meet goals).” Participants were asked to respond to six questions along an eight-item Likert scale ranging from definitely false to definitely true. All factor loadings were above the recommended threshold of 0.40 [40]. The composite reliability of this scale was 0.83 at Follow-up 1 and 0.88 at Follow-up 2.

#### Family functioning

**Figure 2:**
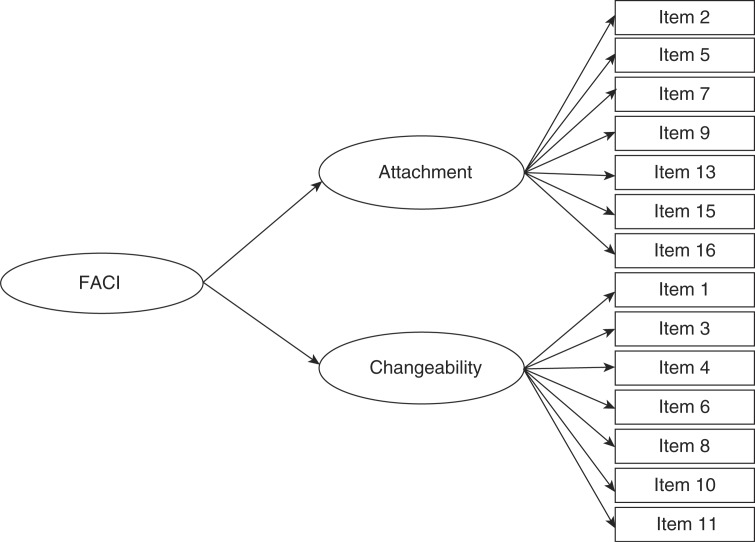
Second-order latent factor of the Family Attachment and Changeability Index (FACI).

The “Family Attachment and Changeability Index” (FACI8) developed by McCubbin, Thompson and Elver [44] consists of 16 items used to measure the family situation of respondents. The items are measured along a five-point Likert scale ranging from always to never. Two of the items returned factor loadings below the 0.40 boundary line (i.e. “It is difficult to get a rule changed in our family” and “Our family tries new ways of dealing with problems”) [40]. These items were not included in the two first-order factors “attachment” and “changeability,” which are indicators of the second-order factor “FACI.” This second-order factor model reflects the theory of McCubbin, Thompson and Elver [44]. Figure 2 offers a graphical representation of the second-order factor, where squares represent the variables measured and ellipses represent the latent variables. The composite reliability of the two first-order factors (Follow-up 1: attachment=0.78, changeability=0.76; Follow-up 2: attachment=0.78, changeability=0.79) and the second-order factor “FACI” at both follow-ups (Follow-up 1=0.78; Follow-up 2=0.84) were above the recommended threshold of 0.70 [41].

#### Control variables

Characteristics of the households in which patients live are captured by information on household size, whether the household head is female, educational level of the household head and real per capita monthly household expenditures (standardized). The socio-demographic characteristics (i.e. age, gender and educational level) of the HIV patients were also taken into account, as well as their physical health (baseline CD4 cell count, current CD4 cell count, presence or absence of side effects, treatment duration in days) and their mental health (anxiety and depression). Other factors included in the analysis are whether patients received nutritional support, whether they used ART intermittently and whether they tried to keep their HIV-positive status secret.

### Analysis

The influence of the PAS on the state of hope in PLWHA was analyzed according to latent cross-lagged modelling. In the analyses, data on patients who completed the first follow-up (*n*=498) were used, because both the PAS intervention and family functioning scale were first measured at this point in time. Using SPSS version 20, the Shapiro–Wilk test of normality indicated that our data deviate from a normal distribution. Subsequent analyses performed with Mplus (version 7) therefore used the MLR estimator robust for non-normal data [45]. The model was estimated conditioned on the observed exogenous variables. Missing data theory applies only to endogenous dependent variables. The adequacy of the different models was evaluated based on Hu and Bentler's cut-off criteria [46], in which two of the following three criteria must be met for a satisfactory global model fit to be attained: comparative fit index (CFI) ≥0.95, root mean square error of approximation (RMSEA) ≤0.06, and standardized root mean square residual (SRMR) ≤0.08.

Measurement equivalence was examined [47–49] using the Chi-square difference test adapted for the MLR estimation [50]. All latent variables were found to be full metric invariant over time. All factors loadings were therefore set as equal across both waves [51]. Furthermore, the measurement error associated with a Follow-up 1 latent factor item is correlated with the measurement error associated with its counterpart item in Follow-up 2 [52]. For the sake of simplicity, the following figures do not include all error-correlation paths. Regression paths between control variables measured at both follow-ups were included in the model [52].

**Figure 3:**
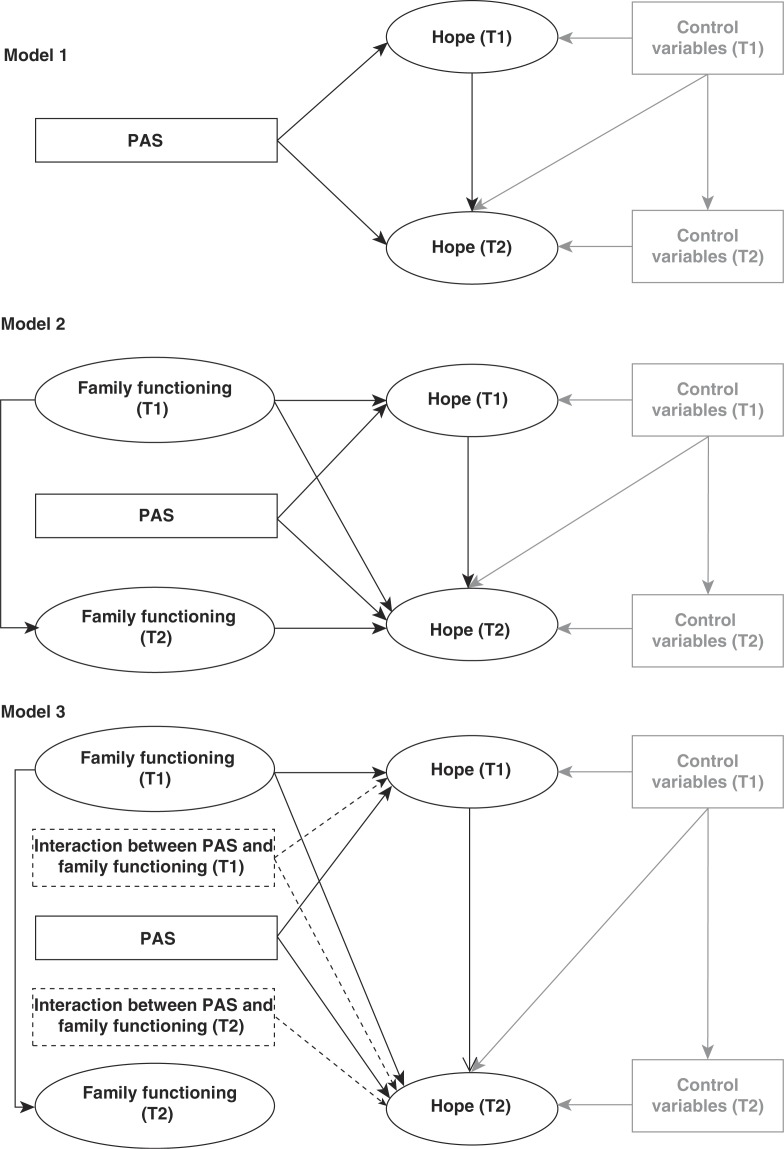
Three models of the impact of the PAS intervention, family functioning and their interaction on state of hope over time.

Figure 3 offers a graphical representation of the models used to analyze the influence of PAS and family environment on the level of hope in PLWHA, as well as to examine the interaction between the two determinants. In order to make this figure less complex, the second-order family functioning factor is represented as a single ellipse. Figure 2 illustrates the second-order factor in more detail. The first model in Figure 3 provides a graphical representation of the first analysis, which examines the impact of the PAS intervention on the latent hope factors by introducing a dummy variable for the intervention. The second model introduces the second-order latent factors measuring family functioning in both waves. The regression paths for the two interaction terms – estimated using the method of Klein and Moosbrugger [53] – between the latent family-function factor and the PAS variable are shown in Model 3.

## Results

Table 1 provides an overview of descriptive statistics for the sample. The results of the three estimated models are displayed in Table 2. The PAS intervention, the family functioning variables and the interaction effects were introduced into the models in a stepwise fashion. For the sake of simplicity in the overview, the autoregressive paths between the time-changing control variables are not shown. The first model in Table 2 shows a reasonable fit (RMSEA=0.045, CFI=0.893 and SRMR=0.049). No significant effects were found for the PAS intervention, whether in the short or medium term. As indicated by the R^2^ scores, 16.5% of the variability in hope is explained by the selected variables at the first follow-up, whereas 22.9% is explained at the second follow-up. The second model explains 16.9% of the variance in the state of hope at Follow-up 1 and 23.5% at Follow-up 2. Model 2 shows a satisfactory global model fit based on the goodness of fit criteria of Hu and Bentler's article [46], which states that the analyses should meet two of the following three criteria: CFI/TLI ≥0.95, RMSEA ≤0.06 and SRMR ≤0.08 [46]. The RMSEA score (0.039) and the SRMR score (0.064) are below the recommended levels (RMSEA ≤0.06, and SRMR ≤0.08) for obtaining a good fit [46], while the CFI score (0.835) does not reach the recommended threshold of 0.95 [46]. The significance of the interaction term indicates that the third model should be preferred over the second model [54]. In the third model, the autoregressive path from the family functioning variable at Follow-up 1 to its equivalent in Follow-up 2 has a coefficient of 0.258 (*p*=0.008). Other aspects, such as adequacy and interpretability of parameter estimates, also remain critical in deciding on the validity of a model [55]. In this regard, parameter estimates have been shown to be good measures of their latent constructs [40] and the composite reliability of the scales appeared to have reliable scales [41]. Furthermore, all latent variables were found to be fully metric invariant over time, which implies that respondents attributed the same meaning to the latent construct over time [51]. Both the adequacy of the measures and the interpretability of the estimates – which should also be taken into account for model evaluation – support the results [56]. In this regard, in line with the socio-ecological framework, the importance of investigating the interrelationship is supported by the significant interaction term between family functioning and the PAS intervention in the final model. This positive interaction effect indicates that better functioning in affected families increases the positive effect of PAS on the state of hope in PLWHA at the second follow-up.

**Table 1:** Descriptive statistics for the sample

Level of hope	Follow-up 1	Follow-up 2
Average sum score of Snyder's state of hope scale	40.30 (*SD*: 7.04)	40.35 (*SD*: 7.21)
Control variables (time constant)		
Sex		
Male	22.6%	
Female	77.4%	
Education level		
No formal education	3.1%	
Primary education	26.9%	
Some secondary education	46.9%	
Completed secondary education	20.0%	
Tertiary education	3.1%	
Age (years)	38.9 (*SD*: 9.5)	
Nutritional support	35.0%	
Treatment duration (days)	504.25 (*SD*: 86.82)	
CD4 cell count at baseline	147.85 (*SD*: 109.71)	
Control variables (time changing)		
CD4 cell count	326.8 (*SD*: 191.1)	371.3 (*SD*: 207.42)
Intermittent use of ART	8.9%	6.9%
Side effects (yes)	8.1%	8.5%
Keep HIV a secret	44.2%	43.7%
Anxiety and depression index	5.82 (*SD*: 5.66)	4.47 (*SD*: 4.92)
Household size	3.2 (*SD*: 1.87)	3.1 (*SD*: 1.90)
Sex of household head (Female)	62.1%	64.3%
Education level of household head		
No formal education	9.4%	9.8%
Primary education	38.2%	36.1%
Some secondary education	38.6%	39.5%
Completed secondary education	11.4%	12.7%
Tertiary education	2.4%	1.9%
Real per capita monthly household expenditure (rand)	867.71 (*SD*: 953.99)	864.15 (*SD*: 1156.46)

**Table 2:** Model results of a cross-lagged regression on state of hope over time (*N*=309)

	Model 1		Model 2		Model 3	
	
	Hope (T1)	Hope (T2)	Hope (T1)	Hope (T2)	Hope (T1)	Hope (T2)
State of hope						
Hope (T1)		0.180*		0.186*		0.207*
PAS	−0.174	−0.127	−0.178	−0.123	−0.187	−0.131
Family functioning						
FACI8 (T1)			0.085	−0.034	0.152	0.205
FACI8 (T2)				0.103		−0.331
Interaction term						
PAS*FACI8 (T1)					−0.122	−0.330
PAS*FACI8 (T2)						0.560*
Control variables (time constant)						
Education level	0.089	0.166*	0.095	0.162*	0.097	0.164*
Age	0.009	0.009	0.009	0.009	0.009	0.007
Sex	−0.147	0.026	−0.143	0.020	−0.148	0.032
Nutritional support	−0.006	−0.129	−0.015	−0.127	−0.010	−0.123
CD4 count at baseline	0.000	0.000	0.000	−0.001	0.000	−0.001
Treatment duration (days)	−0.001*	−0.001	−0.001	−0.001	−0.001	−0.001
Control variables (T1)						
Household size	0.020	0.071	0.025	0.072	0.026	0.069
Sex of household head	−0.013	−0.127	−0.018	−0.131	−0.020	−0.173
Education level of household head	0.007	−0.110	0.004	−0.112	0.004	−0.103
Side effects	−0.096	−0.024	−0.104	−0.021	−0.103	−0.003
Real per capita monthly household expenditure	0.000	0.000**	0.000	0.000**	0.000	0.000**
Keep HIV a secret	0.048	0.017	0.042	0.020	0.044	0.030
Intermittent use of ART	−0.682**	0.228	−0.679**	0.254	−0.680**	0.263
CD4 count	0.000	0.000	0.000	0.000	0.000	0.000
Anxiety and depression	−0.027**	−0.006	−0.026*	−0.005	−0.025*	−0.003
Control variables (T2)						
Household size		−0.053		−0.052		−0.046
Sex of household head		0.159		0.153		0.192
Education level of household head		0.086		0.086		0.084
Side effects		−0.288		−0.292		−0.291
Real per capita monthly household expenditure		0.000		0.000		0.000
Keep HIV a secret		−0.249*		−0.256*		−0.247*
Intermittent use of ART		−0.605*		−0.619*		−0.539*
CD4 count		0.000		0.000		0.000
Anxiety and depression		−0.023*		−0.023*		−0.025*
RMSEA	0.045		0.039			
CFI	0.893		0.835			
SRMR	0.049		0.064			
Chi^2^ (df, *p*)	781.019 (482, *p*=0.000)		2719.483 (1859, *p*=0.000)			
BIC	22951.945		48947.453		48949.189	
AIC	22892.401		48835.667		48835.718	

* ≤0.05

** ≤0.01.

## Discussion

The relative success of the ART scale-up has generated a great deal of hope [18]. It is helping to free countries in sub-Saharan Africa from a cycle of vulnerability in which the HIV epidemic interacts with poverty and other forms of inequality [3,18]. In this study, the level of hope amongst PLWHA was above the neutral point of the scale, indicating that, on average, patients held positive aspirations for the future. Nevertheless, the need for effective interventions to “create environments conducive to securing hope” [18, p. S8] in the long term is underscored by the recent climate of uncertainty about the continuity of funding from large donors [57,58] and the current stock-outs of certain ARV drugs (e.g. Fixed Dose Combination) in some South African provinces [59]. Appropriate psychosocial support is required in order to avoid exposing individuals to these uncertainties. In this regard, the double aim of this article was (1) to analyze the impact of the PAS intervention and the family environment on the state of hope in PLWHA in the Free State and (2) to investigate the interaction between this intervention and the immediate social contexts in which a patient lives. With regard to the first aim, our results indicate that the PAS intervention had no direct effect on the state of hope in PLWHA. This finding underscores the importance of the social context in explaining the discrepant findings on the effectiveness of the PAS intervention and its widely differing effect sizes [26,27]. There is a need for research that incorporates the social units in which community-based adherence counsellors support individual patients [26]. To this end, the family environment was incorporated into the analysis in a second step. Based on the results, this environmental factor also had no significant influence. Building on the socio-ecological perspective, the interrelatedness and interdependency between individuals and their broader social contexts should be taken into account [32–34]. This analysis, which serves the second research objective, reveals that better functioning families are better able to translate PAS into higher levels of hope at the patient level. Acting as catalysts to PAS, such families are more adaptive to new circumstances in their environments, and they act as cooperative entities. The interplay between well-functioning families and external PAS results in higher levels of hope. Hope is an essential dimension of successful coping in the context of HIV/AIDS [12–14] and can also have substantial impacts on behaviour in the present [19]. With higher levels of hope, PLWHA are better able to seek alternative pathways to reaching the goals that they had set before their positive diagnosis. Furthermore, after diagnosis, high-hope patients are better able to generate new goals that are appropriate to their changed health statuses. Hoping and coping are thus inextricably linked [42].

This study has four particular strengths. First, it focuses on hope, which is an under-researched concept in the social sciences with regard to HIV prevention and treatment [18]. Second, it operationalizes hope with structural equation modelling over time, as hope can be seen as a continuum [3], which is also a process [15,60] that can change throughout the course of illness [3,30]. A third strength of this study is that it uses data drawn from a randomized controlled trial in order to investigate the short-term to medium-term effects of PAS interventions. This procedure is in line with the argument of Bernays, Rhodes and Barnett [18] regarding the need to develop a “longer term vision in developing and researching interventions that enable environments that can shape and sustain hope through HIV prevention and HIV treatment engagement.” Finally, the study addresses the context of fragile delivery, as the survey took place immediately after large provincial ART stock-outs in the Free State province in 2008/2009.

In addition to these strengths, however, it is important to note the limitations of our study. First, the absence of a random sample limits the generalizability of the findings. Large-scale longitudinal studies aimed at re-assessing these interrelationships are an important research target. Second, these analyses could not control for non-independence of observations due to nesting in districts and clinics, because the limited number of clusters does not make these analyses possible [61]. Future research should take this into account when designing data collection. Third, although these analyses provide unique insight into the interplay between family functioning and PAS on the state of hope in PLWHA, in-depth qualitative research could be interesting in further investigations involving the nature of the dynamics of hope, including the role of time horizons [3], change through the course of illness [30] and its relationship with despair or hopelessness [16]. Exploring gender differences might be another interesting path for further research. Moreover, future research should pay attention to aspects such as intermittent use of ART, mental health and HIV non-disclosure, as well as how these aspects interact with a patient's social environment to influence hope. Understanding better what influences a patient's feelings of hope is important in responding to the long-term challenges of living with HIV/AIDS.

This study has both theoretical and practical implications. From a theoretical point of view, it introduces the intermediate role of households between the two existing research streams (i.e. the dominant individual-level approach and the more recent community-level approach). Few studies have investigated the level of the family or the household, despite the crucial role played by these levels in the social contexts surrounding patients. An individual seldom lives in isolation from his or her family, society's basic social unit, particularly in the sub-Saharan African context [62]. Consequently, as stated by Iwelunmor and colleagues [63] “in Africa, HIV and AIDS has become a complex collective experience shared by many families and communities across the continent” [63]. Most studies that do address the family context in sub-Saharan Africa attribute a passive role to families. This study shifts the focus, viewing families “as challenged, not as damaged” [64]. In responding to the challenges of HIV/AIDS, families have immense potential for providing strength and support [63]. For instance, a supportive family environment might motivate ART adherence [65] and play an important role in supplying messages of hope [66]. In order to capitalize on the strengths of families, future research and policy programming should explore the potential of family-based interventions that can be used in conjunction with peer support interventions that aim to improve hope, among other objectives. With regard to this active perspective, very few intervention studies have assessed the impact of family dynamics on HIV treatment [67–70]. Moreover, none of these studies were performed in high HIV-prevalence countries of sub-Saharan Africa, where such studies form a high research priority in order to study the impact of ART in a comprehensive manner. Whereas existing research points to the importance of the encompassing social context [26,71–73], this article provides quantitative evidence derived from a randomized controlled trial. From the perspective of practice and policy, these results underscore the need for treatment adherence support interventions that are sensitive to the contexts in which they are implemented [74,75], in order to secure the quality and effectiveness of treatment adherence support in a sustainable manner. These results are in line with the warning of Escott and Walley (2005) that “careful assessment of suitability for community-based treatment, rather than the assumption that it is possible for all, is important but may be neglected in the drive to implement a community-based programme” [76]. This article illustrates the fact that the success of an intervention may be supported or hindered by the patient's surrounding social environment [77]. Consequently, in implementing intervention programmes, attention should be paid to pre-existing social dynamics in order to derive optimal programme outcomes [73]. Inspired by these results, further research and policy programming is required to enable treatment adherence supporters to adequately tailor their activities to the different contexts in which they support patients.

## Conclusions

The interplay between well-functioning families and external PAS generates higher levels of hope, which is an essential dimension in successful coping for the long-term challenges of HIV/AIDS. This study increases our understanding of the important role played by family dynamics in HIV/AIDS care. Furthermore, these results underscore the need for PAS interventions that are sensitive to the contexts in which they are implemented.
